# Prevalence of percutaneous injuries and associated factors among health care workers in Hawassa referral and adare District hospitals, Hawassa, Ethiopia, January 2014

**DOI:** 10.1186/s12889-015-2642-0

**Published:** 2016-01-05

**Authors:** Gudeta Kaweti, Teferi Abegaz

**Affiliations:** 1 Health management information system, Hawassa University, P.O. Box: 1560, Hawassa, Ethiopia; 2 School of public and environmental health, Hawassa University, Hawassa, Ethiopia

**Keywords:** Health care workers, Percutaneous injury, Public hospital, Southern Ethiopia

## Abstract

**Background:**

Accidental percutaneous injury and acquiring blood-borne diseases are common problems among health care workers (HCWs). However, little is known about the prevalence and associated factors for needle stick injury among HCWs in Ethiopia.

**Methods:**

A cross sectional study was conducted by including 526 HCWs (physicians, nurses, laboratory technicians, midwives and others), working in two public hospitals (Hawassa Referral and Adare District hospitals), from January 1–30, 2014. Binary logistic regression was done to assess the association of selected independent variables with accidental percutaneous injury.

**Results:**

The prevalence of at least one episode of percutaneous injury was about 46 % of which more than half (28 %) occurred within one year prior to the study period and only 24 % took prophylaxis for human immune deficiency virus (HIV) infection. The adjusted logistic regression analysis revealed that HCWs who recap needles were twice as likely to face a percutaneous injury. Chance of exposure to needle stick or sharp injuries also increased with increase in educational status. Having a previous history of needle stick or sharp injury was found as one of the risk factors for the occurrence of another injury. Nurses and cleaners were also at increased risk for the occurrence of percutaneous injuries.

**Conclusion:**

Needle stick and sharp injuries were common among HCWs in the study hospitals, which warrants training on preventive methods.

## Background

Health care workers (HCWs) are at increased risk of accidental injury and acquiring infections including hepatitis virus and human immune deficiency virus (HIV) infection [1]. They are also at increased risk of acquiring infection because of direct exposure to patients’ blood and other body fluids [2, 3]. According to World health organization (WHO) report, the annual proportions of HCWs exposed to bloodborne pathogens was 2.6 % for HCV, 5.9 % for HBV and 0.5 % for HIV, worldwide among which the majority was from developing regions (i.e. 40-65 % of HBV and HCV infections in HCWs were attributable to percutaneous occupational exposure) [4, 5]. One study also indicated that 16.000 HCV, 66,000 HBV and 1,000 HIV infections may occurred in the year 2000 worldwide among HCWs due to their occupational exposure to percutaneous injuries [6]. Recognizing this threat, a series of procedures (standard precaution methods) are proposed to prevent occupational exposures and handle potentially infectious materials.

Percutaneous injury may result in serious health risks including psychological trauma, chronic diseases, and even death [6, 7]. Other studies have also shown that occupational exposure to blood through percutaneous injury is a serious health issue among HCWs [2, 8, 9]. Worldwide, thousands of HCWs can be exposed to percutaneous injury per day [2, 4]. As a result, the safety of health care work force and handling complications related to occupational exposure, is a global health concern [2].

In developing countries, the risk of injuries at work place is higher than that of developed countries [4, 10]. Percutaneous injuries are obviously more dangerous for HCWs from developing countries, because work related blood borne pathogens are more prevalent in low-income countries of the world, specifically endemic in sub Saharan African countries [4, 11]. In Rift valley provincial hospital of Kenya, the prevalence of accidental exposures to blood and needle stick injuries was common [12]. Previous studies in northern, southern and eastern parts of Ethiopia have shown the increased risk of occupational blood exposure [2, 10, 13]. Needle stick injury was also reported to be about 31 % among HCWs of Hawassa [10].

Although there is a national guideline on infection prevention, little is known about the risk of exposure and preventive actions. The purpose of this study was to estimate the prevalence of percutaneous injuries and associated risk factors among HCWs in the study area.

## Methods

### Study design, settings and participants

A cross sectional study on percutaneous injuries was conducted among HCWs in Hawassa University Referral and Adare District hospitals. Hawassa University Referral Hospital has 350 beds for admitted patients and is expected to serve 10 to 12 million people of the southern region and the surrounding Oromia zones. Adare District Hospital has 70 functional beds. The study included all HCWs in the study hospitals. Those HCWs who were on official leave during the study period were excluded.

### Data collection

Data collection tool was developed after reviewing different literature. Finally, we adopted the questionnaire from a previous cross sectional study conducted in Dire Dawa administration council and Harari region, Ethiopia, 2010. After adoption, a pretest was done on 20 HCWs at Shashemene Referral hospital. All questionnaire items were translated to Amharic to test for clarity and consistency. Confusing or misleading questions/concepts were modified after the pre test. Data collectors (One senior diploma nurse and one senior BSC laboratory professional) were trained and assigned for data collection process.

Data collection was conducted from January 1–30, 2014 during working day, on tea break and by appointment. Filled questionnaires were checked on daily bases for completeness and clarity. Close supervision was conducted by principal investigator. Data collectors and supervisors reached study participants through getting permission.

### Measurements of injuries

Dependent variable was one year and ever history of percutaneous injury. Outcome assessment was based on answers to the questions on the number of percutaneous injuries the participant had experienced during their entire career and one year prior to the study. Each factor was dichotomized into and coded by giving 0 to the group hypothesized as having a lower risk and 1 to the group hypothesized as having a higher risk.

The independent variables included age, gender, educational status; employment/qualification, department, personal protective practices, knowledge and attitude related questions. Those potential risk factors for percutaneous injuries were selected basd on reviewing previous literatures.

### Statistical analysis

Accuracy of data was checked timely, and data cleaning was made before analysis. Collected data was entered into epi-data software, exported to Statistical Package for the Social Sciences (SPSS) version 16, cleaned and analyzed. Percutaneous injury is dichotomized in two ways before analysis, ever and one year percutaneous injury. The dichotomization was done to simplify analysis and interpretation of the results. Association between dependent and independent variables was examined using bivariate and multivariate logistic regression models and reported as unadjusted odds ratio (OR) and adjusted odds ratios (AOR) with 95 % confidence interval (CI). P-value was set at less than 0.05 to verify existence of association. In order to avoid an excessive numbers of variables and unstable estimates, only variables that reached a P-value less than 0.25 were included in the subsequent analysis (multivariate logistic regression analysis model).

### Ethical clearance

Ethical clearance was obtained from institutional review boards (IRB) of both Hawassa University College of medicine and Health sciences and Addis Continental Institute of public Health. Written consent was obtained from Hawassa referral and Adare district hospitals. All study participants were informed about the importance of the study and finally verbal consent was obtained before data collection. Participants had the right to refuse participation or terminate their involvement at any point during the study. Information obtained from each respondent was kept confidential. Any section of report writing did not refer to a specific respondent.

## Results

**Fig. 1:**
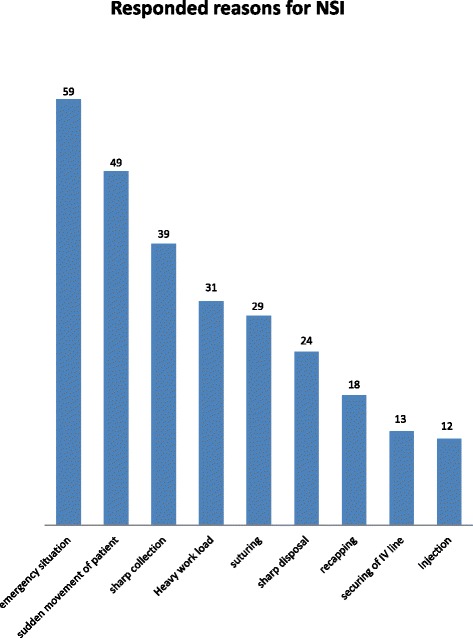
Self reported reasons for needle stick injuries among HCWs who ever faced a needle stick injury. Emergency situation, sudden movement of the patient, sharp collection, heavy work load, suturing, sharp disposal, recapping needles, securing of IV line and injection were the main reported reasons for the occurrence of both needle stick and sharps injuries

Of the 526 eligible HCWs, 496(94.3 %) were consented and completed the questionnaire. The mean age of study respondents was 28.4 (SD = ±6.7) years. The demographic characteristic of the study participants is presented in Table 1. Of these, 46 % were exposed to percutaneous injuries in their professional life and 28 % of them faced injury one year prior to the study. Among exposed HCWs, only 24 % took anti-HIV infection prophylaxis. Different reasons were reported for the needle stick injuries. Emergency situation (28.6 %), sudden movement of the patient (23.8 %) and sharp collection (18.9 %) were the top three reported reasons for the occurrence of needle sticks injury followed by work overload, suturing, waste disposal and needle recapping respectively (Fig. 1)

**Table 1:** ocio demographic characteristics of respondents by sex, age, educational status and departments, Hawassa, Southern Ethiopia, 2014 (*N* = 496)*

SVariables		Hawassa Referral Hospital (%)	Adare District Hospital (%)	Total (%)
Sex	Male	128 (25.81)	33 (6.65)	161 (32.5)
	Female	274 (55.24)	61 (12.30)	335 (67.5)
Age	15-24	93 (18.75)	29 (5.85)	122 (24.6)
	25-34	230 (46.37)	55 (11.09)	285 (57.5)
	35-44	65 (13.10)	7 (1.43)	72 (14.5)
	>44	14 (2.82)	3 (0.6)	17 (3.4)
Educational status	Below diploma**	126 (25.4)	21 (4.23)	147 (29.6)
	Diploma***	125 (25.2)	46 (9.27)	171 (34.5)
	Degree and above****	151 (30.45)	27 (5.44)	178 (35.89)
Department	Outpatient department	152 (30.65)	46 (9.27)	198 (39.9)
	Inpatient department	147 (29.64)	20 (4.03)	167 (33.7)
	Delivery and operation room	73 (14.72)	17 (3.43)	90 (18.1)
	other@	30 (6.05)	11 (2.22)	41 (8.3)

*Mean age of respondents by year: Mean ± SD = 28.43 ± 6.7

Other@ = includes ENT, Dermatology, Dental unit, Oncology unit

**Have educational status with no certification on specific skill

***Trained on specific subject and certified with that specific skill, but with educational status of below bachelor of science/art

****Includes those who holds bachelor of science/, master of science/art, PhD, and above

**Table 2:** Multivariate logistic regression analysis result for percutaneous injury, Hawassa, Southern Ethiopia, 2014 (*N* = 496)

Variables		Percutanous injury		Crude OR (95 % CI)	Adjusted OR (95 %)
		Yes	No		
		N (%)	N (%)		
Age	15-24 year	44 (36.1)	78 (63.9)	1	1
	25-34 year	136 (47.7)	149 (52.3)	1.62 (1.05-2.50)*	0.81 (0.45-1.47)
	>34 year	46 (51.7)	43 (48.3)	1.90 (1.09-3.31)*	1.32 (0.60-2.87)
Educational status	Below Diploma	52 (35.4)	95 (64.6)	0.43 (0.27-0.67)***	0.17 (0.06-0.52)**
	Diploma	74 (43.3)	97 (56.7)	0.60 (0.39-0.91)*	0.34 (0.18-0.63)**
	Degree and Above	100 (56.2)	78 (43.8)	1	1
Qualification/type of employment	Physicians	34 (61.8)	21 (38.2)	5.32 (2.56-11.05)***	3.12 (0.97-9.99)
	Nurse/midwife	112 (52.8)	100 (47.2)	3.68 (2.11-6.43)***	4.68 (1.97-11.14)***
	Laboratory Prof	12 (30.8)	27 (69.2)	1.46 (0.63-3.37)	1.13 (0.35-3.71)
	Cleaners	47 (47)	53 (53)	2.91 (1.56-5.45)**	7.45 (2.89-19.15)***
	Others@	21 (23.3)	69 (76.7)	1	1
Department	outpatient departments	84 (42.4)	114 (57.6)	1	1
	Inpatient Departments	78 (46.7)	89 (53.3)	1.19 (0.79-1.80)	0.61 (0.33-1.12)
	Delivery and Operation theatre	49 (54.4)	41 (45.6)	1.62 (0.98-2.68)	1.16 (0.60-2.24)
	Other	15 (36.6)	26 (63.4)	0.78 (0.39-1.57)	0.41 (0.17-1.00)
Additional responsibility	Yes	55 (59.1)	38 (40.9)	1.96 (1.24-3.11)**	1.28 (0.68-2.41)
	No	171 (42.4)	232 (57.6)	1	1
Experience	<2 years	10 (25.6)	29 (74.4)	0.40 (0.19-0.85)*	0.68 (0.26-1.75)
	2-4 years	92 (48.7)	97 (61.3)	1.10 (0.76-1.60)	1.49 (0.91-2.43)
	>4 years	124 (46.3)	144 (53.7)	1	1
Working ≥ 40 hours	Yes	199 (48.2)	214 (51.8)	1.93 (1.17-3.17)*	1.32 (0.71-2.45)
	No	27 (32.5)	56 (67.5)	1	1
Knowledge	Sufficient	157 (43.9)	201 (56.1)	1	1
	Insufficient	69 (50)	69 (50)	1.28 (0.86-1.90)	1.45 (0.88-2.39)
Recapping needle	Yes	103 (53.1)	91 (46.9)	1.65 (1.15-2.37)***	2.15 (1.33-3.49)**
	No	123 (40.7)	179 (59.3)	1	1
Ever BBFs exposure	Yes	92 (65.2)	49 (34.8)	3.10 (2.06-4.66)***	3.02 (1.77-5.15)***
	No	134 (37.7)	221 (62.3)	1	1
Always follow standard. precautions	Yes	102 (42.7)	137 (57.3)	1	1
	No	124 (48.2)	133 (51.8)	1.25 (0.88-1.79)	1.18 (0.75-1.88)
Always use glove for procedure	Yes	179 (45.8)	212 (54.2)	1	1
	No	13 (28.9)	32 (71.1)	0.48 (0.25-0.95)*	0.59 (0.26-1.34)

Note:

✓ model classification accuracy is =69.7 %

✓ *p < 0.05, **p < 0.01 and ***p < 0.001

✓ The categorical reference is selected based on scientifically meaningful manner

The odds of injury among HCWs who recap needle was about two times (AOR = 2.15; 95 % CI: 1.33 - 3.49) more compared to those who did not recap. Age and sex were not the predictors of percutaneous injury (Table 2). On the other hand, compared to others (laundry staff, porters, sample transporters, health officers and anesthetists), working as nurse (AOR = 4.68; 95 % CI: 1.97-11.14) and cleaners (AOR = 7.45; 95 % CI: 2.89- 19.15) has significantly increased the risk of percutaneous injury. HCWs with a previous history of splash exposure were about 3 times at increased risk of facing percutaneous injuries (AOR = 3.02; 95 % CI: 1.77- 5.15) as compared to those who have no history of such exposure. In this study, there was 83 % less odds of injury among HCWs who had educational status of below diploma (AOR = 0.17; 95 % CI: 0.06- 0.52) and 66 % less odds of injury among HCWs who had educational status of diploma (AOR = 0.34; 95 % CI: 0.18, 0.63).

The association was highly significant in both groups (Table 2). Statistically, there is no significant difference among study participants with regard to variation in age, service year, additional responsibilities, working more than 40 h/week, and difference in knowledge status, gloving practice, as well as whether they think that they are always following standard procedure or not.

In this study 48.2 % of HCWs reported that they regularly follow standard procedures and regression model indicated that regularly following standard precautions has no significant relation with percutaneous injury. About 83.3 % of the participants reported dissatisfaction by the provision of infection prevention materials, 73 % of whom cited lack of supply (scarcity) as the main reason. Lack of infection prevention supplies seriously affects prevention efforts and puts patients, visitors and HCWs at greater risk of infection and adds to the dissatisfaction of HCWs with their work environment. Furthermore, 73.6 % of the HCWs perceived their work place to have put them at higher risk of acquiring HBV, HCV and HIV infections and 41.1 % preferred treating HBV, HCV and HIV patients separate from other patients. About 78 % of HCWs worry when caring for patients having blood borne pathogens such HBV, HCV and HIV. About 92 % of HCWs knew that contaminated needles can transmit HIV. Almost 44 % of the participants in our study responded that recapping contaminated needles immediately after use can prevent accidental injury. Few numbers or one tenth of study participants dispose sharps and needles in safety box and less than one third of HCWs in two hospitals had ever attended work place training on injuries.

## Discussion

In this study, nearly half of HCWs had experienced at least one percutaneous injury during their entire career and more than a quarter had experienced injury one year prior to the study. Percutaneous injury was significantly associated with needle recapping, qualification, educational status and history of previous exposure. The prevalence of percutaneous injuries observed in this study was slightly higher than estimates in earlier Ethiopia and African studies [2, 10, 14]. The difference could be explained by the fact that our study population included non health professionals who have less knowledge on exposure prevention strategies and consequences of exposure.

Reported one year needle stick injury - alone was 26.6 %, which is lower than study conducted in Hawassa city [10], University of Gondar [13] and Uganda [15], but higher than one year needle stick injury report from Harari Regional State and Dire Dawa Administrative Council [2]. Needle stick injury was around 60 % in a Greek general hospital [16]. In this study, 92 % of HCWs knew that contaminated needles can transmit HIV. This report is almost similar with report from Nigeria [14]. About 83 % of HCWs reported dissatisfaction by the supply of infection prevention and control materials. This is about twice as high as found in a study conducted among HCWs in eastern Ethiopia 44.8 % [2].

In this study, needle stick recapping contributed more to the occurrence of percutaneous injuries. Those HCWs who recapped needles were at a significantly increased risk of sustaining such injury compared to those who didn’t recap, which is consistent with studies done in Uganda [15], Iowa community hospital [17], and Tehran [9]. Almost 44 % of the participants in our study responded that recapping contaminated needles immediately after use can prevent accidental injury, which contradicts the standard. Any used needles and sharps are recommended to be disposed in safety box. Few or about one tenth of study participants dispose sharps and needles in safety box while about one third of HCWs belief that glove and gown were not required for each contact with patients.

Generally, a higher percentage of respondents in this study, as well as in prior studies in Nigeria [14], Turkey [18], southern Ethiopia [10] and eastern Ethiopia [2] had risky practice of needle recapping. Recapping of needles, not using protective glove and improper usage of safety box are malpractices [2, 7] that could be improved by training.

Less than one third of HCWs in two hospitals had ever attended work place training on injuries.

This implies that most HCWs depend on their experience and previous knowledge they have acquired from school.

After combining nurses and midwifery, there was about 5 times increased risk of sustaining a percutaneous injury compared to others/laundry staff, porters, health officers and anesthetist/. Workload and working for more than 40 h/week were previously identified reasons for exposure of nurses and midwives [12, 15]. Higher percentage of participants and respondents in this study, as well as in prior studies in a provincial hospital of Kenya [12], in Turkey [19], and in a tertiary care hospital of Pakistan [20] were nurses which may contribute for this finding.

Cleaners were 7 times at increased risk of percutaneous injury compared to laundry staff, porters, health officers and anesthetists. The confidence interval is somewhat wider which indicates that further research with a bigger sample size is mandatory. However injuries are common during disposal of waste in another report as well [21], which may be the main reason for this association. Less knowledge may be another reason, because out of the cleaners who participated in the study, 80 % did not get any infection prevention and control related training prior to the research.

According to this finding, being exposed will increase the risk of repeated exposure by 3-fold. Even though we did not come up with evidences on this specific finding, it seems that HCWs who had previously been exposed will ignore the safety procedures and consider exposure as normal. Probably, their first exposure did not bring a harsh outcome to them. In this study, why an increase in educational status is a risk factor for the occurrence of injuries could not be explained. We could not as well find previous studies showing this association. Further studies are highly warranted to verify this association.

## Limitations

This study has its own limitations. Based solely on the data from two hospitals is not strong enough to give inferences to the HCWs population in other hospitals. Because of the voluntary participation into the study, some degree of selection bias could not be ruled out, as those who had got percutaneous injuries might have been more eager to participate. This could lead to some overestimation of injury rate; even if it would not affect the relations observed with the risk factors, as general population was taken. If any selection according to risk factors would have taken place, it is likely that the participation had been more active among those who were interested in training and precautionary measures, and thus, the observed relation might slightly underestimate the true risks. As this is a cross sectional study, the limitations that come with this type of design need to be taken into consideration when interpreting the findings. The study response rate was 94.3 % which is much higher compared to other studies [1, 7, 13] and no particular characteristic could be identified in non respondents except that some HCWs were unavailable as they had either joined short courses, enrolled to a higher institute for further study or were on leave. So in general, we think that our results are likely to reflect quite well what was happening among HCWs.

## Conclusion

In conclusion, there is a high prevalence of both needle sticks and sharps injuries in the study areas. We detected suboptimal practices and behaviors that put both patients and HCWs at significant risk of acquiring occupational infections. Recapping practice, educational status, qualification and history of previous exposure were identified as important predictors of percutaneous injuries. Top management bodies of the study areas need to improve the training of HCWs and to provide infection prevention materials. Regular reporting, follow up and assessment of occupational exposures need to be carried out in health institutions. Additional study involving rural and central hospitals is highly recommended for further evidence.

## Abbreviations

AOR: adjusted odds ratio
HBV: hepatitis B virus
HCV: hepatitis C virus
HCWs: health care workers
CI: confidence interval
HIV: human immunodeficiency virus
OR: odds ratio
SD: standard deviation
